# 

**Published:** 1856-04

**Authors:** 


CONTENTS—ORIGINAL COMMUNICATIONS.

The Dental Student's Preparatory Education :—An Adress. By Rev. B. P. AYDELOTT, D. D. .... 221
Minutes of the Fourth Annual Meeting of the Ohio Dental College Association..... 229
Twelfth Annual Meeting of the Mississippi Valley Association of Dental Surgeons.. 236
Inflammation of the Dentine. By J. HAMILL.... 252
Report of Discussions of the Missisippi Valley Association.... 259
Case of Hypertrophy of the Gums, in the Practice of Professor GROSS. Reported by WILLIAM H. GODDARD, D. D. S., Louisville, Ky.... 276
Valedictory Address to the Graduates of the Ohio College of Dental Surgery, of the Session of 1855-6. By PROF. J. TAFT.... 282

SELECTED ARTICLES.

On the Metal Aluminum. By H. L. BURPEE, Brooklyn.... 295
Local Anæsthesia.... 299
The Paris Exhibition.... 300
Pivoting Teeth. By JOHN COGHLAN, M. D., M. R. C. S., ENG.... 305
Amalgam for Filling Teeth.... 308
Quackery. By J. W. SALISBURY, M. D.... 319
Baneful Effects of Saleratus and Cream of Tartar.... 322
Miscellaneous Items.... 326
Correspondence—Original.... 327
Editorial....2.... 330

